# A comparison of light-harvesting performance of silicon nanocones and nanowires for radial-junction solar cells

**DOI:** 10.1038/srep11532

**Published:** 2015-06-26

**Authors:** Yingfeng Li, Meicheng Li, Pengfei Fu, Ruike Li, Dandan Song, Chao Shen, Yan Zhao

**Affiliations:** 1 State Key Laboratory of Alternate Electrical Power System with Renewable Energy Sources, North China Electric Power University, Beijing, 102206, China; 2 Chongqing Materials Research Institute, Chongqing, 400707, China

## Abstract

Silicon nanorod based radial-junction solar cells are competitive alternatives to traditional planar silicon solar cells. In various silicon nanorods, nanocone is always considered to be better than nanowire in light-absorption. Nevertheless, we find that this notion isn’t absolutely correct. Silicon nanocone is indeed significantly superior over nanowire in light-concentration due to its continuous diameters, and thus resonant wavelengths excited. However, the concentrated light can’t be effectively absorbed and converted to photogenerated carriers, since its propagation path in silicon nanocone is shorter than that in nanowire. The results provide critical clues for the design of silicon nanorod based radial-junction solar cells.

Silicon nanorod can introduce fundamental improvements in optical and electrical properties for photovoltaic applications^1^,^2^,^3^,^4^,^5^,^6^,^7^. Firstly, the single silicon nanorod can act as Nano-antennas via the leaky radial modes excitation and thus have outstanding light-harvesting ability, which will reach several times of that calculated from the Lambert-Beer law^6^,^7^,^8^. Secondly, unique radial-junction (RJ) can be fabricated in silicon nanorod, allowing orthogonalization of light absorption direction to charge carrier collection^2^,^9^. As a consequence, silicon nanorod RJ solar cells are considered as low-cost alternatives to the traditional planar silicon solar cells^10^. As of today, the highest efficiency of silicon nanorod RJ solar cell has reached 17.11% already^11^.

In the reported silicon nanorod RJ solar cells, the ones based on silicon nanocone (SiNC) always perform better than those based on silicon nanowire (SiNW). For example, solar cells based on SiNC can output a doubled power conversion efficiency than those based on SiNW^12^,^13^. This phenomenon is always explained by the better light-absorption ability of SiNC, due to its continuous diameter thus effective reflective index^14^,^15^,^16^. However, we think this conclusion needs to be further discussed, since it is not draw out based on comparing the optical performance of SiNC and SiNW arrays composed of the equivalent amount of silicon material.

In this work, we investigate the optical characteristics of SiNC and SiNW owing the same volume, by reliable theoretical simulations^17^,^18^. The SiNW is modeled as a circular cylinder with hemisphere top, and the SiNC is modeled by five equilong subwires, as shown in Fig. 1a,b. Through comparing the light-concentration and light-absorption abilities of them under the same surface coverage rate, it has been demonstrated that SiNC shows no advantage over SiNW in light-absorption for RJ solar cells. Such result provides a critical clue for optimizing SiNC based photovoltaic devices.

## Results and discussion

**Figure 2:**
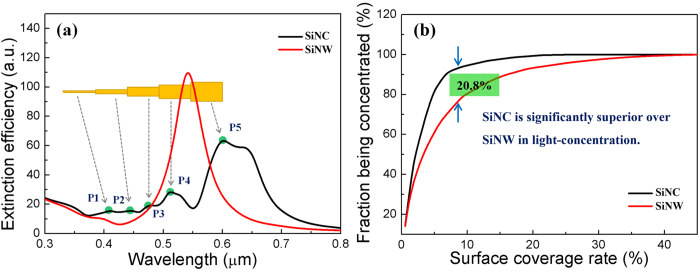
(**a**) Extinction efficiency spectra of monomer SiNC and SiNW composed of equivalent amount of material. (**b**) The fraction of sunlight being harvested by the SiNC and SiNW array, under different surface coverage rates.

The extinction efficiency curves of the monomer SiNC and SiNW were obtained, as given in Fig. 2a. Obviously, the extinction efficiencies of SiNC are higher than those of SiNW in most of the wavelengths. This implies that the monomer SiNC owns greater broadband light-concentration ability than SiNW, according to the definition of extinction efficiency^17^, Q_ext_ = C_ext_ /πr^2^, where C_ext_ and πr^2^ represents the extinction and geometric cross section of the target respectively.

To estimate the light-concentration ability of the SiNC and SiNW array, firstly, we calculate the extinction efficiency of them by
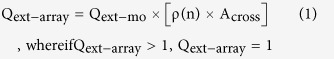


Q_ext-mo_ is the extinction efficiency of monomer as shown in Fig. 2a, ρ(n) is the surface number density, and A_cross_ is the monomer cross-sectional area. A_cross_ of SiNC is also taken as the real geometric cross-sectional area of the SiNW to coincide with the calculation of Q_ext-mo_ (not given). This equation should be generally reliable since the light-harvesting abilities of semiconductor nanocone and nanowire array are both insensitive to the pitch between adjacent rods^20^.

Then, we weight Q_ext-array_ by solar-spectrum *AM1.5* and integrate it over waveband 300–800 nm. Finally, the obtained integral is divided by the total amount of incident light in the same waveband, and the fraction of sunlight being harvested by the SiNC and SiNW arrays is gained. The obtained fractions as a function of the surface coverage rates are plotted in Fig. 2b. Notably, the SiNC array shows significant superiority over SiNW array in light-concentration. Especially under surface coverage rate ~8.5%, the SiNC array can harvest 20.8% more sunlight than SiNW array.

This superiority of SiNC over SiNW array in light-concentration can be explained by its continuous diameters and thus resonant wavelengths excited. As shown in Fig. 2a, the five peaks emerging in the extinction curve of SiNC correspond to the five subwires with different diameters respectively. Through calculating the extinction spectra of the five subwires respectively, we have marked the owner-member relationships between the peaks and the subwires.

**Figure 3:**
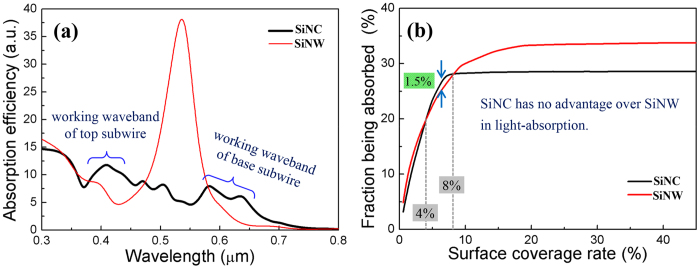
(**a**) Absorption efficiency spectra of monomer SiNC and SiNW. (**b**) The fraction of sunlight being absorbed by the SiNC and SiNW arrays, under different surface coverage rates.

However, the superior light-concentration ability not necessarily implies SiNC is better than SiNW in light-absorption for RJ solar cells. This is because that, there have two outcomes for the light being concentrated by the target: to be absorbed and to be scattered out. Of them, only the part being absorbed can be utilized to generate carriers, which is significative for RJ solar cells. The absorption efficiency spectra of the monomer SiNC and SiNW are given in Fig. 3a.

It is notable that the absorption efficiency curve of SiNC displays very different shape from its extinction one in Fig. 2a. At the working waveband of the top subwire, SiNC still shows obvious superiority over SiNW in light-absorption, similar as in light-concentration. While at the working waveband of the base subwire, the superiority of SiNC over SiNW in light-absorption shrinks very significantly comparing with that in light-concentration. As a consequence, SiNC shows no obvious advantage over SiNW in light-absorption, despite its significant superior light-concentration ability.

The light-absorption ability of SiNC and SiNW arrays is also estimated, by calculating the fraction of sunlight being absorbed by them, with the same strategy used above. From Fig. 3b, it can be intuitively seen that, under most of the surface coverage rates, the light-absorption performance of the SiNC arrays is worse than that of the SiNW arrays. Only under the surface coverage rates ranging from 4% to 8%, the SiNC arrays exhibit slight advantage over the SiNW arrays in light-absorption. The biggest difference (occurring under the surface coverage rate 6.6%) is merely about 1.5%, which is quite smaller than the counterpart value in light-concentration, 20.8%. So, it can be concluded that SiNC shows no better performance than SiNW in light-absorption for RJ solar cells application.

**Figure 4:**
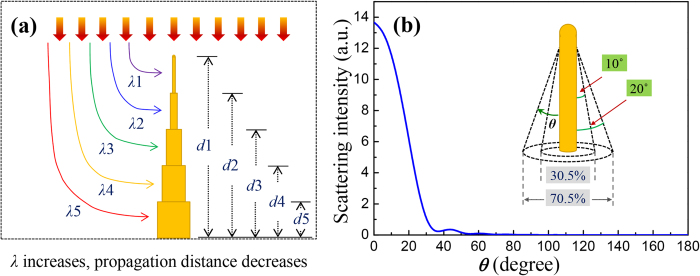
(**a**) Schematic view of the resonance wavelength of every subwire in the SiNC, and corresponding propagation distance of the light they concentrated. (**b**) Angle distribution of the scattering light around the SiNW at its resonance wavelengths.

The gradually decaying light-absorption ability of SiNC with increasing wavelength can be attributed to that, in SiNC, the light concentrated by the lower subwires owns shorter propagation distance, as illustrated in Fig. 4a. Since the resonance wavelengths of the subwires increase from the top to the bottom of the SiNC, the propagation distances of them decrease gradually. According to the Lambert-Beer law, this means the light with long wavelength can’t be as effectively absorbed as that with short wavelength. Such analyses give a reasonable explanation for the fact that the huge advantage of SiNC in light-concentration at the waveband corresponding to P5 (Fig. 2a) shrinks dramatically in light-absorption (Fig. 3a). This explanation may not be suitable for nanostructures composed of material of high light absorption coefficient, whose light-absorption ability is not so sensitive to the light propagation distance. Thus GaAs^21^ and α-Si^15^ nanocones both show enhanced light-absorption ability than corresponding nanowires.

We have also analyzed the reason for the better performance of SiNC than SiNW in light-absorption at short waveband, by investigating the light scattering properties of the SiNW. As shown in Fig. 4b, the angle distribution of the scattering light around the SiNW at its resonance wavelength is plotted. An axial localization feature can be obviously observed that, 70.5% of the scattering light will be confined in the cone angle of 20°. This means, in SiNC, parts of the scattering light from the upper subwires may be harvested again by the below ones.

In summary, through comparing the optical response features of SiNC and SiNW (monomer and arrays) composed of the same amount of silicon materials, we find that SiNC has significant superiority over SiNW in light-concentration, which indicates that SiNC arrays should be a perfect light-trapping structure for bulk silicon solar cells. However, the light concentrated by the SiNC can’t be absorbed effectively.

For RJ solar cells application, only the light being absorbed in the SiNC can be converted into free charge carriers, which is of significance. Therefore, it can be concluded that SiNC is not better than SiNW in light-absorption for RJ solar cells. The worse light-absorption in SiNC is mainly due to that, the light in SiNC owns shorter propagation distance than in SiNW. The axial localization feature of the scattering light partly contributes to the better performance of SiNC than SiNW in light-absorption for short wavebands.

## Methods

### Theoretical simulations

Extinction and absorption spectra are calculated using the discrete dipole approximation (DDA) method^17^, whose reliability on silicon nanostructure has been tested in our previous work^18^. The total lengths of them are both set as 1 μm, with which the solar cells reported perform well^22^. The diameter of the SiNW is set to be 83 nm, corresponding to the optimized light-trapping effect^23^. The SiNC owns the same volume as the SiNW thus its five subwires own diameters of 40, 60, 80, 100 and 120 nm, from the top to the bottom. In the DDA calculation, the SiNC and SiNW are both replaced by cubic point dipoles, whose size has been carefully tested and set as 3 × 3 × 3 nm finally. The iterative precision for the dipole moments has also been tested and set to be 0.001%, to ensure the calculation reliability. Bulk values of the complex index of refraction for silicon are used^24^. Only the incident light from the top is considered, since the optical performances of silicon nanorod is insensitive to incident angle^7^.

## Additional Information

**How to cite this article**: Li, Y. *et al.* A Comparison of Light-harvesting performance of silicon nanocones and nanowires for radial-junction solar cells. *Sci. Rep.* **5**, 11532; doi: 10.1038/srep11532 (2015).

## Figures and Tables

**Figure 1:**
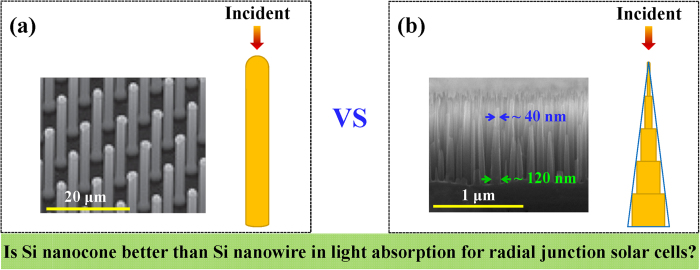
(**a**) The schematic diagram of the SiNW used to model the real geometry of the SiNW fabricated^19^, which has a hemisphere-like top. (**b**) The schematic diagram of the SiNC used to model the SiNC fabricated by us. The inset in (**a**) is adapted by permission from Macmillan Publishers Ltd: Nature Materials, copyright 2010.
